# Supplementary figures and images for: Dpp/TGFβ-superfamily play a dual conserved role in mediating the damage response in the retina
Source: PLoS One. 2021 Oct 26;16(10):e0258872. doi: 10.1371/journal.pone.0258872 (PMC8547621; doi:10.1371/journal.pone.0258872)

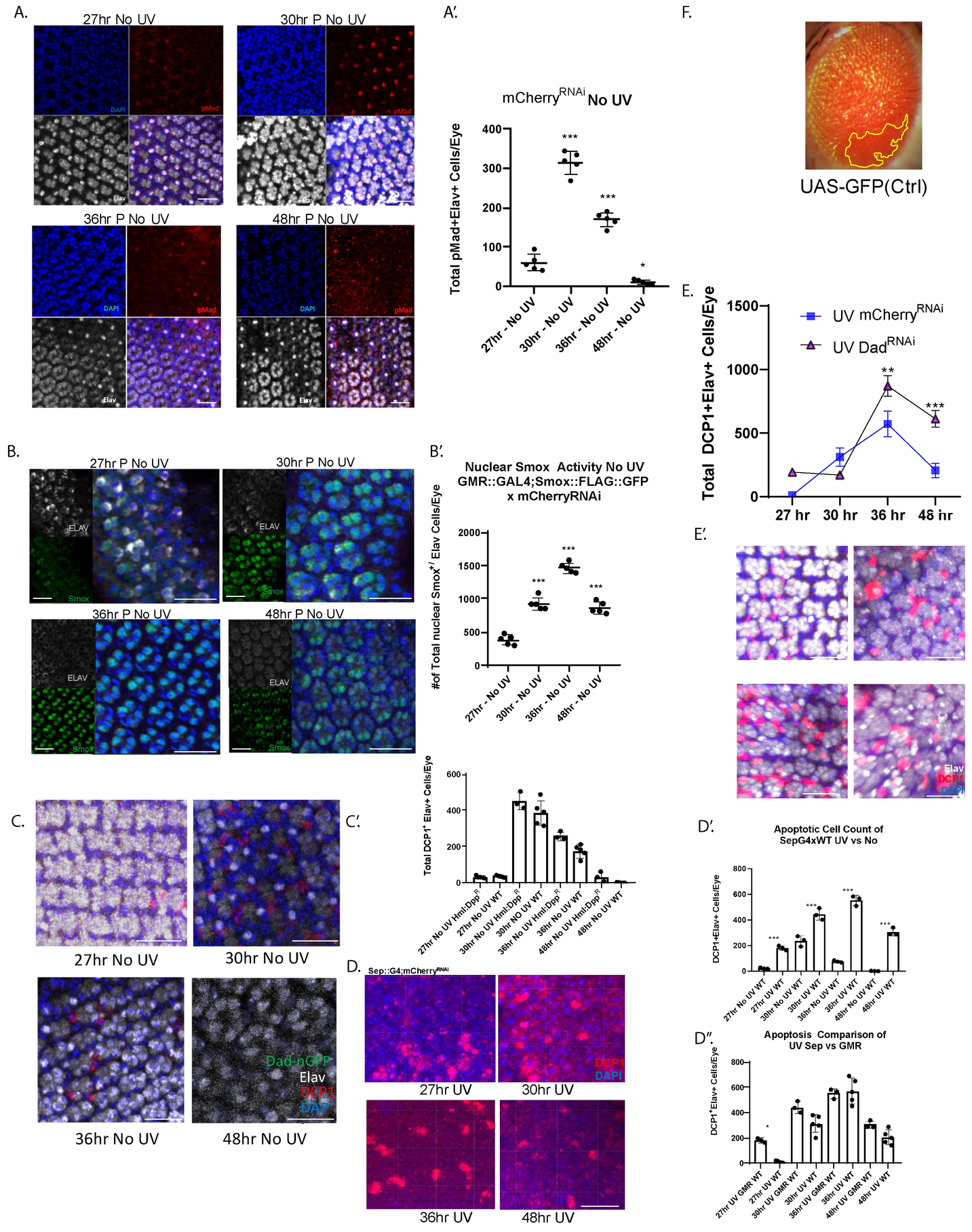

Supplement: S1 Fig — Representative images and quantitation of control pMad+ Elav+ cells under all timepoints and non-UV WT genotype is compared (S1-A). Control nSmox+ Elav+ cells under all timepoints and non-UV WT genotype is compared (S1-B). GMR::Gal4;Hml::DppRNAi progeny total DCP1+ Elav+ cells is compared to control no UV animals (S1-C). Sep::Gal4;mCherry-RNAi control progeny is compared vs no UV and with GMR::Gal4 control progeny (S1-D). GMR::Gal4;UAS::Dad-RNAi is compared with control UV progeny (S1-E). Sep::Gal4;UAS-HepACT; UAS-GFP representative control image (S1-F). Scale Bar: 20 μm. Error bars indicate s.e.m.; P-values from Student’s t-test. *p<0.05, **p<0.01, **p<0.001. (TIF) [file pone.0258872.s001.tif]

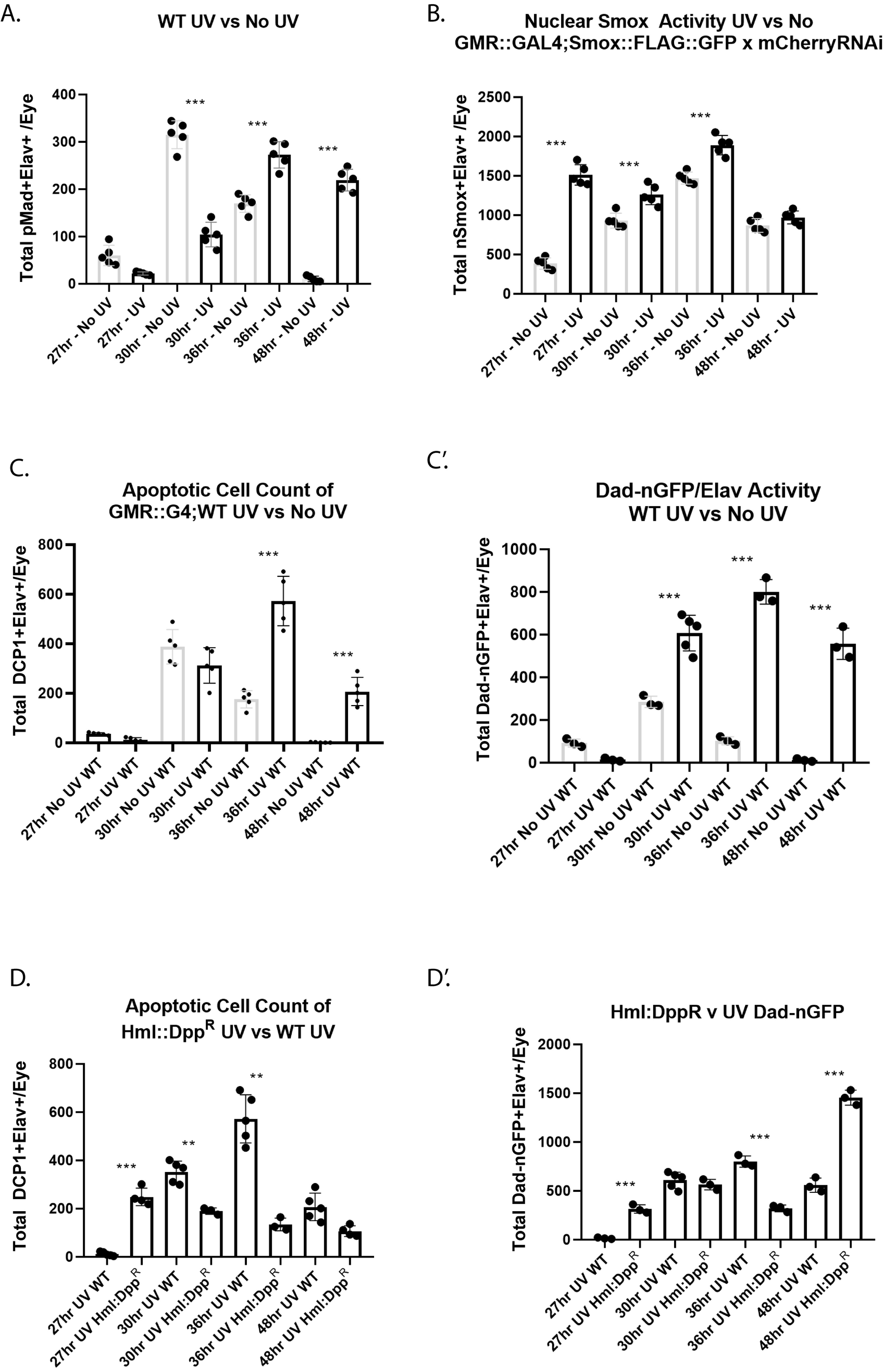

Supplement: S2 Fig — Representative images and quantitation of all experiments were collected below. Control UV WT progeny pMad+ Elav+ cells compared with non UV progeny (S2-A). UV exposed control nSmox+ Elav+ cells is compared with non UV progeny (S2-B). Total WT DCP1+ Elav+ cells quantitation compared with no UV (S2-C). Total Dad-nGFP+ Elav+ cells compared between UV and no UV WT progeny (S2-C’). Individual quantitation of total GMR::G4;Hml::DppRNAi DCP1+ Elav+ cells and Dad-nGFP+ Elav+ cells compared with WT UV (S2-D). Error bars indicate s.e.m.; P-values from Student’s t-test. *p<0.05, **p<0.01, **p<0.001. (TIF) [file pone.0258872.s002.tif]

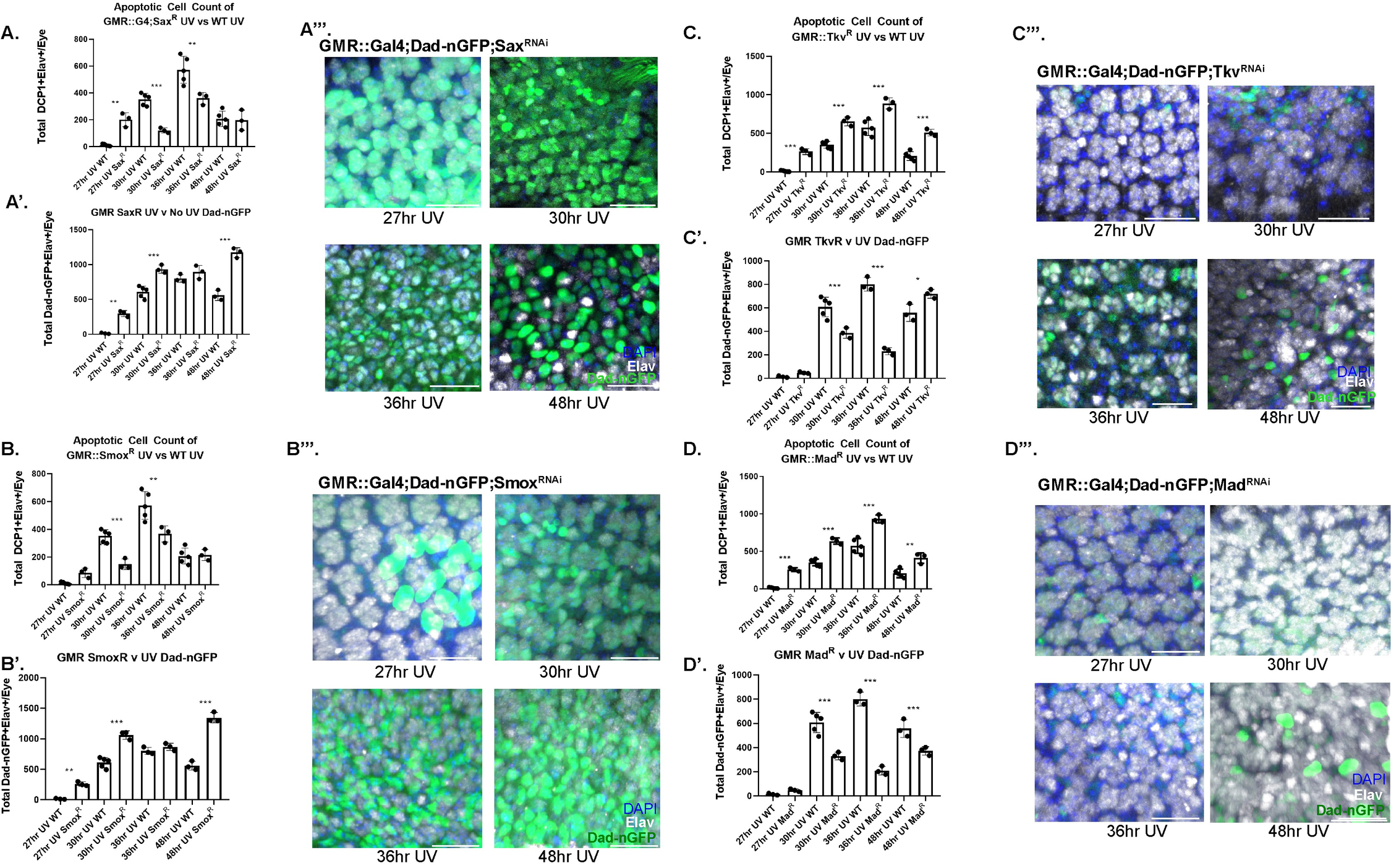

Supplement: S3 Fig — Representative images and quantitation of all experiments were collected below. UV exposed GMR::G4;SaxRNAi individual progeny DCP1+ Elav+ cells and Dad-nGFP+ Elav+ cells are compared to UV WT (S3-A). UV exposed GMR::G4;SmoxRNAi individual progeny DCP1+ Elav+ cells and Dad-nGFP+ Elav+ cells are compared to UV WT (S3-B). UV exposed GMR::G4;TkvRNAi individual progeny DCP1+ Elav+ and Dad-nGFP+ Elav+ cells are compared to UV WT (S3-C). UV exposed GMR::G4;MadRNAi individual progeny DCP1+ Elav+ and Dad-nGFP+ Elav+ cells are compared to UV WT (S3-D). Scale Bar: 20 μm. Error bars indicate s.e.m.; P-values from Student’s t-test. *p<0.05, **p<0.01, **p<0.001. (TIF) [file pone.0258872.s003.tif]

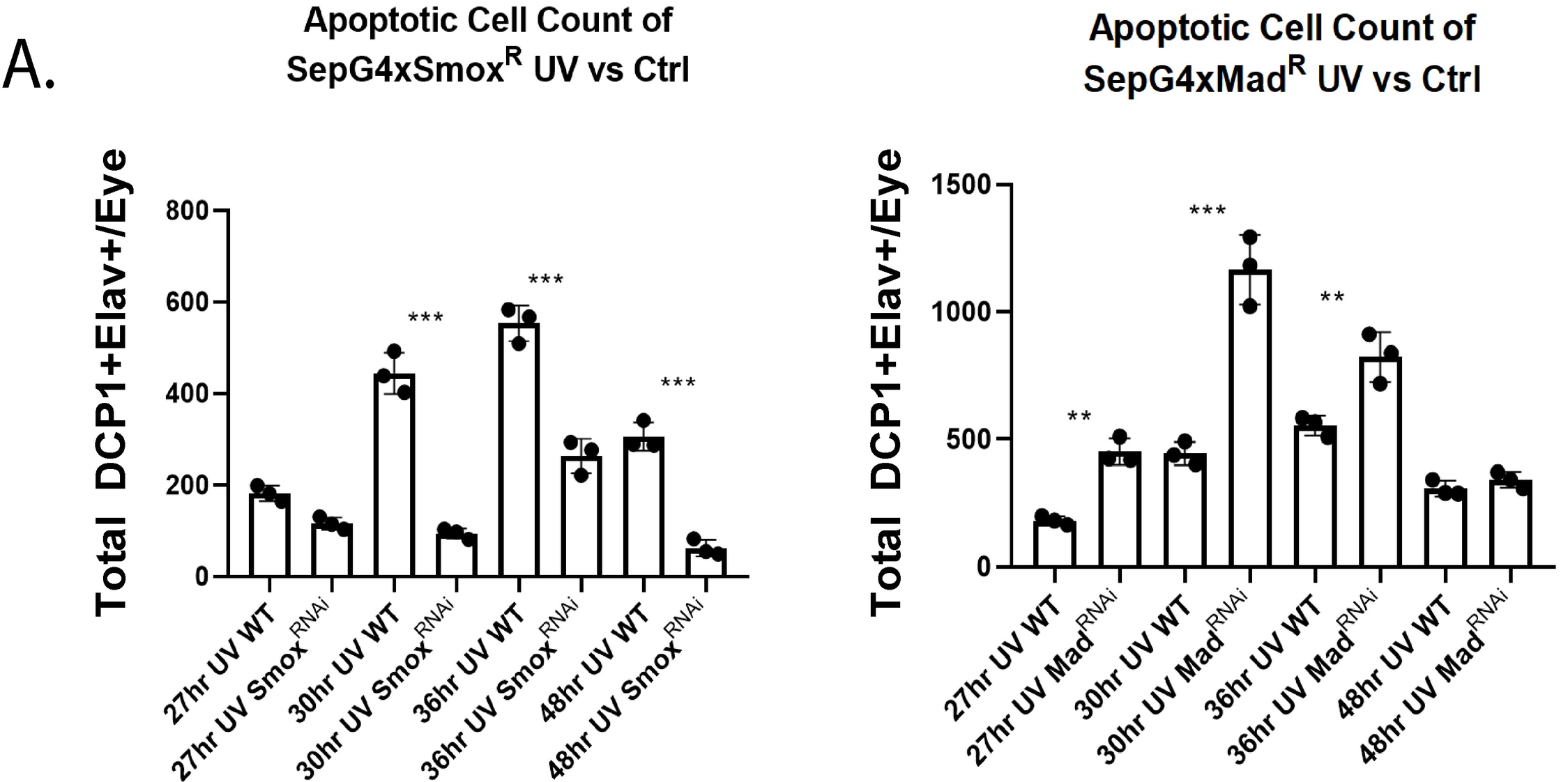

Supplement: S4 Fig — Error bars indicate s.e.m.; P-values from Student’s t-test. *p<0.05, **p<0.01, **p<0.001. (TIF) [file pone.0258872.s004.tif]

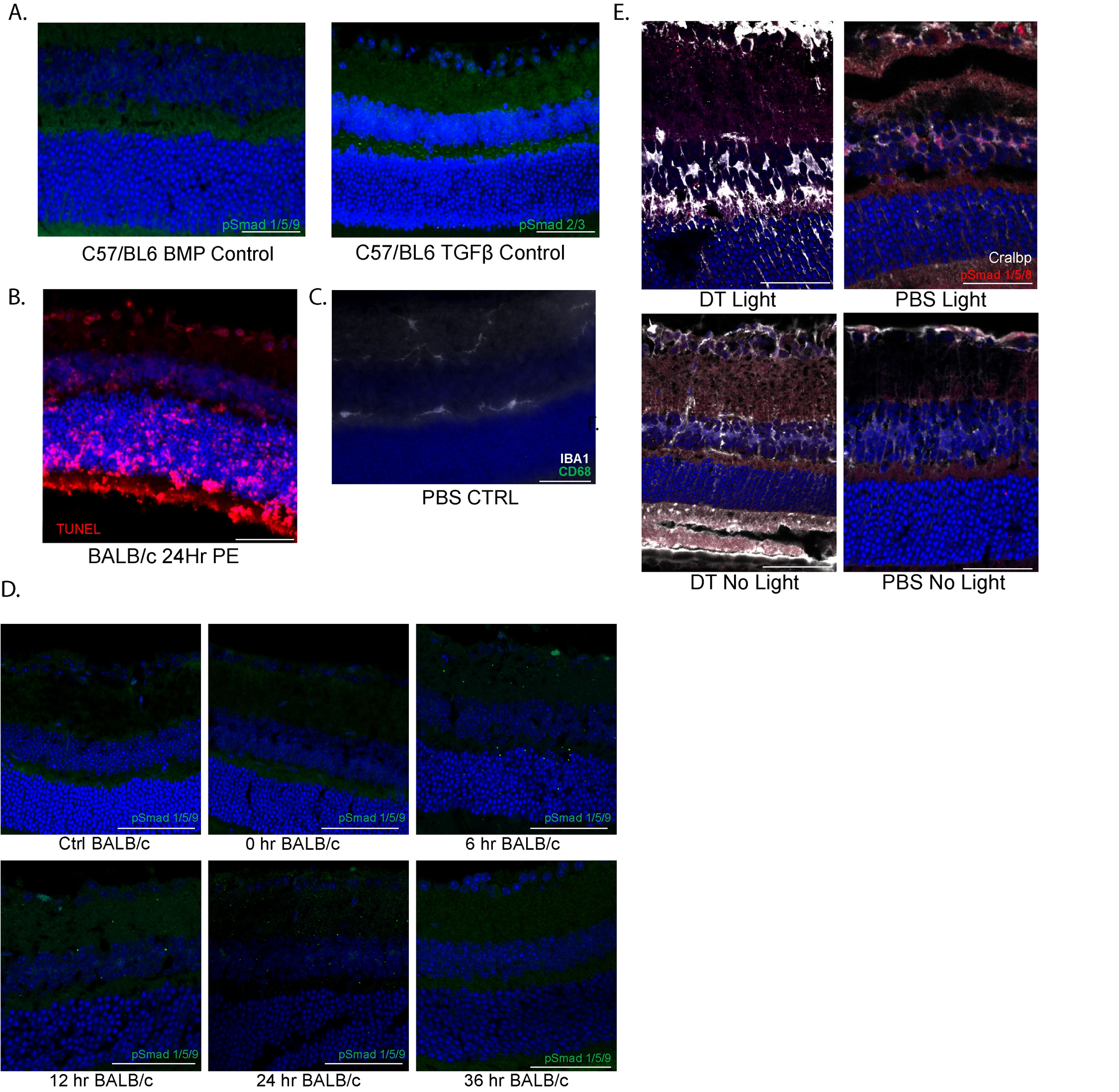

Supplement: S5 Fig — pSmad 1/5/9 (BMP) and pSmad 2/3 (TGFB) control images of C57 animals (S5-A). TUNEL stain in red of BALB/c animals post light exposure, with DAPI in blue (S5-B). PBS WT Control unexposed retina with IBA1 in white and CD68 in green (S5-C). pSmad 1/5/9 representative images of BALB/c animals post light exposure at 0, 24 and 36 hours (S5-E). Representative images of DT injected CD11b::DTR mice with pSmad 1/5/9 (BMP) in red, and CRALBP in white (S5-E). Scale Bar: 30 μm. (TIF) [file pone.0258872.s005.tif]
